# Bulgecin A: The Key to a Broad-Spectrum Inhibitor That Targets Lytic Transglycosylases

**DOI:** 10.3390/antibiotics6010008

**Published:** 2017-02-22

**Authors:** Allison H. Williams, Richard Wheeler, Constance Thiriau, Ahmed Haouz, Muhamed-Kheir Taha, Ivo G. Boneca

**Affiliations:** 1 Institut Pasteur, Unité Biologie et génétique de la paroi bactérienne, Dept. Microbiologie, 28 Rue du Dr. Roux, 75015 Paris, France; richard.wheeler@pasteur.fr (R.W.); constancethiriau@hotmail.com (C.T.); 2 Institut National de la santé et de la Recherche Médicale (INSERM), Groupe Avenir, 75015 Paris, France; 3 Institut Pasteur, CNRS-UMR3528, Plate-forme de Cristallographie, 25 Rue Dr. Roux, 75724 Paris, France; ahmed.haouz@pasteur.fr; 4 Institut Pasteur, Unité des Infection Bactériennes Invasives, Dept. Infection et Epidémiologie, 28 Rue du Dr. Roux, 75015 Paris, France; mktaha@pasteur.fr

**Keywords:** peptidoglycan, *Neisseria*, bulgecin, lytic transglycosylase, beta-lactam

## Abstract

Lytic transglycosylases (Lts) are involved in recycling, cell division, and metabolism of the peptidoglycan. They have been understudied for their usefulness as potential antibacterial targets due to their high redundancy in Gram-negative bacteria. Bulgecin A is an *O*-sulphonated glycopeptide that targets primarily soluble lytic tranglycosylases (Slt). It has been shown that bulgecin A increases the efficacy of β-lactams that target penicillin bindings proteins (PBPs). Here, we present the high-resolution crystal structure of LtgA from *Neisseria meningitidis* strain MC58, a membrane bound homolog of *Escherichia coli* Slt, in complex with bulgecin A. The LtgA-bulgecin A complex reveals the mechanism of inhibition by bulgecin A at near atomic resolution. We further demonstrate that bulgecin A is not only a potent inhibitor of LtgA, but most importantly, it restores the efficacy of β-lactam antibiotics in strains of *N. meningitidis* and *Neisseria gonorrhoeae* that have reduced susceptibility to β-lactams. This is particularly relevant for *N. gonorrhoeae* where no vaccines are available. This work illustrates how best to target dangerous pathogens using a multiple drug target approach, a new and alternative approach to fighting antibiotic resistance.

## 1. Lytic Transglycosylases (Lts) as New Antibiotic Targets

It has been clear since the 1960s that treatment of bacterial infections with β-lactams has consequently given rise to highly resistant bacteria. Since the 1990s, there has been a surge in antibiotic resistance with no new viable targets identified [1]. The problem of antibiotic resistance has been compounded by the lack of discovery of new classes of broad-spectrum antibiotics. The response to the antibiotic resistance crisis has led to the creation of new synthetic analogs that are largely based on existing treatments [2]. Current dogma suggests that β-lactams, the most widely used class of antibiotic, disrupt the delicate balance that occurs between penicillin binding proteins (PBPs) and lytic transglycosylases (Lts) [3]. Recently, it was demonstrated that β-lactams do much more than just inhibit PBPs, but also disable the peptidoglycan (PG) biosynthetic machinery with deleterious outcomes for the bacteria [3]. The effectiveness of β-lactams as a treatment against bacterial infections revolutionized medicine. This success is threatened by the alarming rise of resistance to β-lactam antibiotics.

The peptidoglycan (PG) in Gram-negative bacteria is a thin sacculus composed of glycan strands cross-linked by short stem peptides that surround the cell. PG is crucial in maintaining bacterial shape and rigidity against osmotic pressure, and enzymes involved in the biosynthesis and remodeling of the PG are amongst the best targets for antibiotics [2]. Lts are intricately involved in the metabolism of the PG. These enzymes cleave the β-1,4-glycosidic bond between the N-acetylmuramic acid (MurNAc) and N-acetylglucosamine (GlcNAc) residues [4,5,6,7,8,9,10]. They are space-making autolysins that permit the insertion of newly synthesized PG material during growth, remodeling, and recycling of the cell wall [4,5,6,7,8,9,10]. *Escherichia coli* recycles a majority of their PG fragments, however, both *N. meningitidis* and *N. gonorrhoeae* release cytotoxic PG fragments that are detected by the host [11,12]. *N. gonorrhoeae* releases more PG fragments than other Gram-negative bacteria, including other *Neisseria* species [13]. Released PG fragments are known to induce a Nod1-dependent inflammatory response [14,15,16,17]. Blocking the release of cytotoxic PG fragments could be a novel path toward anti-virulence development. This approach could have a profound impact on the treatment of Gram-negative pathogenic infections.

Similar to PBPs, Lts are highly redundant enzymes. *E. coli* has eight Lts, seven that are membrane bound (MltA, MltB, MltC, MltD, MltE, MltF, MltG) and one that is a soluble periplasmic enzyme (soluble lytic tranglycosylase (Slt)70) [18]. *Neisseria* species have five identified Lts (LtgA, LtgB, LtgC, LtgD, and LtgE) that have been previously characterized [19,20,21]. Recent studies have started to unravel the functional reasons for the redundancy of Lts across Gram-negative bacteria. For example, it is known that deletion of *ltgA* and *ltgD* in *N. gonorrhoeae* eliminates the release of cytotoxic PG monomers [11,20]. More recently, it was revealed that LtgA primarily localizes at the septum, while LtgD is localized around the cell. This suggests that LtgA produces PG monomers that are largely taken into the cytoplasm for recycling, and LtgD produces PG monomers that are released [22]. Additionally, MltE, a membrane bound Lt from *E. coli*, was recently shown to be involved in the assembly of the type VI secretion system (T6SS) in enteroaggregative *E. coli* [23]. MltE in the presence of an inhibitor, bulgecin A, affects the function of the T6SS, suggesting a more specialized role in the bacteria cell wall [23].

Lts engage in protein-protein interactions with enzymes involved in PG synthesis, such as PBPs, amidases, and PG hydrolases [18]. Although the precise role of Lts in these PG assemblies is unclear, altogether, these enzymes sustain growth and division of the bacteria without disrupting the structural integrity of the cell wall [24,25,26,27,28]. To date, nearly all the Lts from *E. coli* have been structurally characterized. Lts have distinct overall secondary structural differences, however, the catalytic domain, architecture of the active site, enzymatic activities, and substrate specificities are uniquely conserved [29]. This conservation makes Lts natural targets for broad-spectrum inhibitors that target their enzymatic activities.

**Figure 1:**
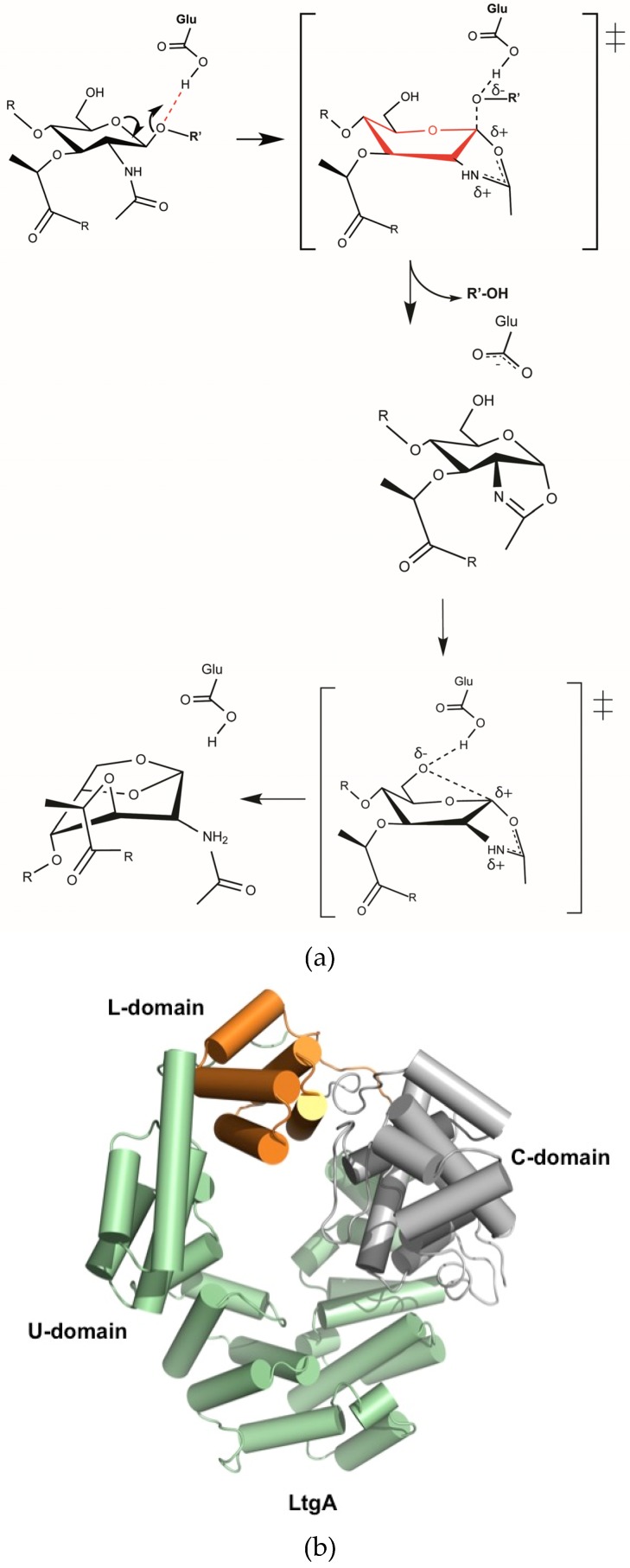
(**a**) Proposed catalytic mechanism of lytic transglycosylases (Lts). Lts use a single catalytic glutamate or aspartate in a simple acid and then base catalysis. In the absence of a second catalytic residue in the active site, these enzymes proceed via anachimeric assistance of the N-acetylmuramic acid (MurNAc) 2-acetamido group and formation of the oxazolinium ion intermediate [31]; (**b**) Overall structure of LtgA. LtgA has three domains: U (green), L (orange) and C (grey). Structure is displayed in a front view. The helices are represented as cylinders.

Based primarily on the crystal structures of Slt70 and Slt35, it is proposed that Lts utilize a single catalytic residue playing the role of an acid and then a base (Figure 1a) [9,29,30,31,32]. Mechanistically, Lts are known to use a single catalytic glutamate or aspartate in a simple acid then base catalysis (Figure 1a). In the absence of a second catalytic residue in the active site of Lts, these enzymes likely proceed via anachimeric assistance of the MurNAc 2-acetamido group and formation of the oxazolinium ion intermediate [31]. Previous studies deciphering the mechanistic details of Lts revealed that β-hexosaminidase inhibitor (NAG-thiazoline) inhibits the activity of Lts. This seminal study was the first to establish a direct link between the formation of 1,6-anhydromuramoyl and the oxazolinium ion intermediate [31]. In the second half of the mechanism, the catalytic glutamate acts as a general base to abstract the proton from the C-6 hydroxyl, leading to its nucleophilic attack on the anomeric center and the formation of a 1,6-anhydromuramic acid product [31].

It has been demonstrated that bacteria devoid of Slts are sensitive to β-lactams and display a bulging phenotype prior to bacteriolysis [33]. The same effect is seen when β-lactams are combined with bulgecin A, an *O*-sulfonated glycopeptide with known antibacterial activity toward Slt70 [33,34]. Previous structural and biochemical evidence shows that bulgecin A specifically targets Slts from *E. coli*. The synergism between β-lactams and bulgecin A has previously been demonstrated in *E. coli*, *Helicobacter pylori*, *Pseudomonas aeruginosa*, and *Acinetobacter baumannii* [33,34,35,36].

In *Neisseria* species, specifically, *N. meningitidis* and *N. gonorrhoeae*, reduced susceptibility to β-lactams is mediated by polymorphism of the *penA* gene (encoding PBP2) [37,38]. Penicillin, the most widely used β-lactam, binds PBPs and inhibits their transpeptidase activity. Modifications in the transpeptidase active site of PBPs results in reduced efficacy of penicillin towards PBPs [37,38]. Patients infected with *N. meningitidis* and *N. gonorrhoeae*, particularly penicillin-resistant strains, could benefit from targeting Lts and PBPs simultaneously in a dual therapy approach.

To evaluate the potential of bulgecin A as a broad-spectrum inhibitor for Lts, we solved the high-resolution crystal structures of the membrane bound Lt (LtgA) from the *Neisseria* species in complex with bulgecin A. The near atomic resolution structure of the LtgA-bulgecin A complex shows the complete structure of bulgecin A supported by a well-defined solvent model that clearly shows highly conserved residues interacting directly with bulgecin A and those that are engaged via water mediated interactions. Bulgecin A appears to mimic the oxocarbonium intermediate step in the predicted mechanism of Lts and is most likely competitive with the glycan strand of the PG. The enzymatic, biological, and structural evidence shows that bulgecin A inhibits LtgA. Furthermore, we demonstrate that bulgecin A, combined with β-Lactams, could be a useful alternative strategy for restoring the efficacy of β-lactams in resistant strains of *N. meningitidis* and *N. gonorrhoeae*. This study will facilitate the design of potent, broad-spectrum inhibitors that target Lts.

## 2. Results

### 2.1. The Native Structure of LtgA

The native structure of LtgA closely resembles its *E. coli* Slt70 counterpart. We have determined the crystal structure of LtgA lacking the signal sequence (MKHSLPLLAALVLAACSSTN) necessary for its membrane localization (Table 1 and Figure 1b). LtgA is responsible for the release of cytotoxic PG monomers from *Neisseria* pathogenic species [11,21]. LtgA shares approximately 33% similarity in the C-terminal domain to its soluble periplasmic *E. coli* counterpart, Slt70, but is predicted to be an outer-membrane lipoprotein that is localized to the septum of *N. gonorrhoeae* [9,22] (Figure 1b). Lts belong to the Family 1 of glycoside hydrolases (GH) family 23, and shares sequence similarity to the goose-type lysozyme. Based primarily on the classification of Blackburn and Clarke, family 1 is further subdivided into five subfamilies (1A to 1E). Slt70 and LtgA both belong to family 1A.

LtgA is a “heart-shaped” highly alpha-superhelical structure consisting of 37 α-helices (Figure 1b). LtgA has three distinct domains: the C-domain, which is the catalytic domain, and the L and U-domains for which the function is unknown (Figure 1b and Figure S1a). The C-domain (colored in grey) is connected to the L-domain by a linker, but is firmly held in place by the U (colored in green) and L (orange) domains. Unlike the structure of Slt70, the U domain (colored in green) partially overlaps with the L-domain (colored in orange) and completely locks this structure (Figure 1b and Figure S1a,b).

### 2.2. Bulgecin A Occupies the Active Site of LtgA

**Figure 2:**
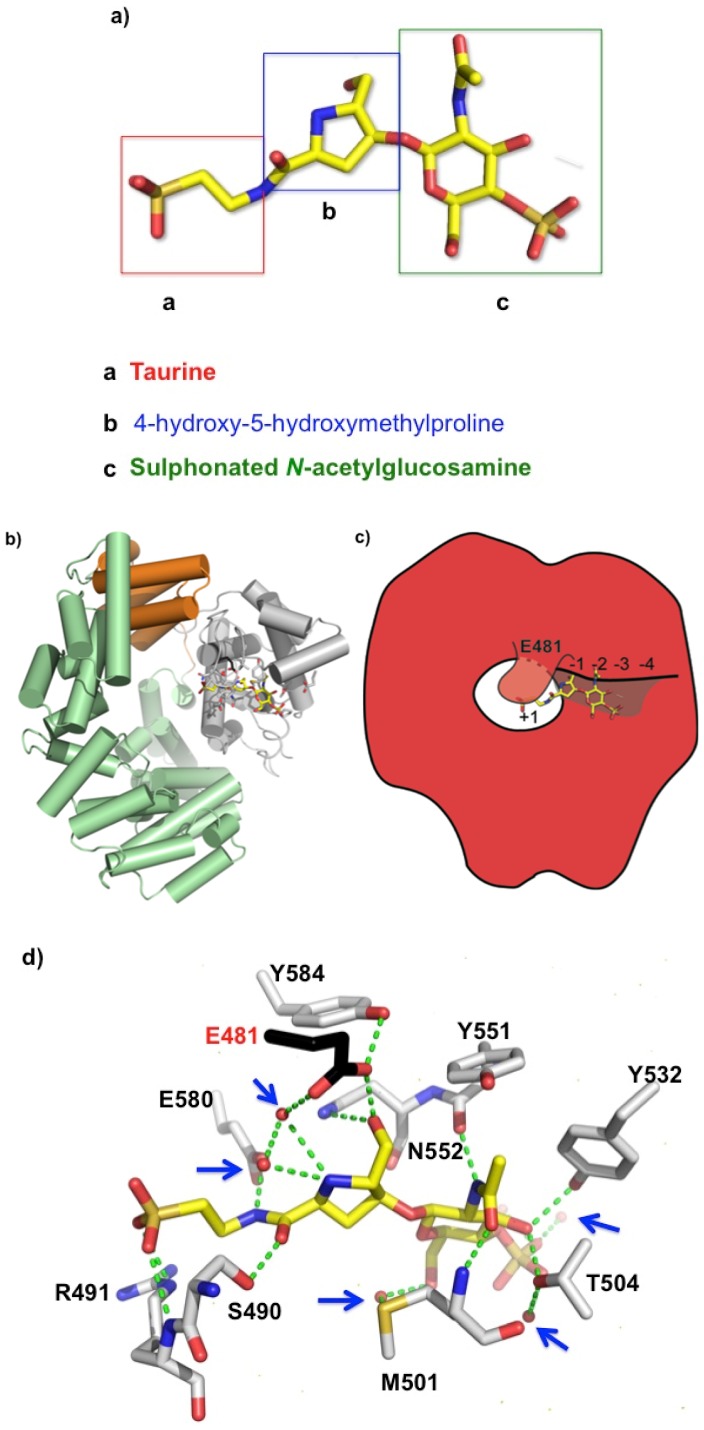

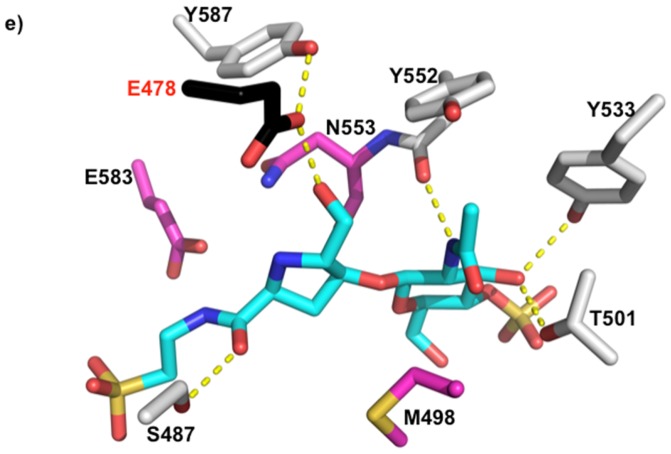
(**a**) Bulgecin A, a sulphonated *N*-acetyl-d-glucosamine unit linked to a 4-hydroxy-5-hydroxymethylproline ring by a β-glycosidic linkage. Crystal structure of LtgA in complex with bulgecin A at 1.78 angstroms; (**b**) Domain organization of LtgA in which the helices are represented by cylinders, and the active site residues are illustrated in a stick model. Active site residues are mainly colored in grey and catalytic residue in black for contrast. Bulgecin A is colored in yellow. In this front view, bulgecin A (yellow) is binding in the putative active site of LtgA; (**c**) Schematic of bulgecin A in the active site of LtgA; (**d**) Noncovalent interactions between bulgecin A and conserved residues in the active site of LtgA (Figure S2 alignment). Water molecules are represented by red spheres and further highlighted with blue arrows; (**e**) Close-up of the active site of bulgecin A in the active site of Slt70. For contrast to Figure 2d, bulgecin A is colored in blue. Conserved residues that are making interactions in the LtgA-bulgecin A complex are colored in pink. In the soluble lytic tranglycosylases (Slt)70-bulgecin A complex a solvent model is undefined because it is at lower resolution 2.8 angstroms [35].

Bulgecin A consists of a 4-*O*-sulfonyl-*N*-acetylglucosamine moiety that is linked by a β 1-4 glycosidic linkage to a 4-hydroxy-5-(hydroxymethyl)-l-proline (Figure 2a) that terminates with a taurine (*N*-linked sulfur) moiety. The density for the complete structure of bulgecin A is clear and consistent in the active site of LtgA (Figure S2). Bulgecin A is bound in the region of the active site defined as subsite −1, −2, −3, and partially occludes subsite +1 (Figure 2b,c). The inhibitor is perfectly poised to block the PG glycan strand. An alignment of LtgA catalytic domain with Slt70, MltE, and MltC demonstrates absolute conservation of the active site of LtgA (Figure S3). A docked chito-oligossacharide (chitopentaose) in the active site of LtgA mimics the PG glycan strand (Figure S4). In the active site of LtgA, chitopentaose and bulgecin A have overlapping binding sites (Figure 2b–d and Figure S4). This strongly suggests that bulgecin A could be competitive with the PG glycan strand. The 4-*O* sulfonated group of the GlcNAc residue forms hydrogen bonds through a well-defined solvent model that was not observed in the Slt70-bulgecin structure, because this complex was at a lower resolution (2.8 angstroms) [35] (Figure 2d). The sulfate partially occludes subsite −3 that would block access to the LtgA active site and possibly contribute to the dose-dependent inhibition of bulgecin A. The O42 hydroxyl of the sulfur makes an indirect interaction via a water molecule with T504, and is the primary contact of the sulfur group with the active site of LtgA. The sulfate is bound to two additional water molecules that form a continuous network of hydrogen bonds in the active site. These water molecules surrounding bulgecin A in the active site of LtgA potentially contribute to inhibitor stability and efficacy and may be important in substrate binding and catalysis (Figure 2d). There are five water molecules that are in direct contact with bulgecin A. Three are involved in water-mediated interactions with residues in the active site of LtgA.

The GlcNAc residue of bulegecin A is well supported by a network of anionic interactions in the active site of LtgA. In subsite −2, the GlcNAc residue forms direct hydrogen bonds with the side chains of Y532 and T504 and with the main chain of M501 and Y551 (Figure 2d). The GlcNAc residue is involved in a water-mediated interaction with M501 (Figure 2d). In subsite −1, the hydroxyl-methyl of the proline is directly hydrogen bonded to the OE2 of the catalytic residue E481 and the ND2 of N552. The N1 of proline makes a water mediated interaction with the OE1 of the catalytic residue E481. The backbone carbonyl group of the proline is directly hydrogen bonded to S490. The taurine group of the inhibitor is directly hydrogen bonded to S490 and E580. The OS1 and OS2 of the sulphonated portion of the taurine is directly hydrogen bonded to the main chain and side chain of R491. R491 is unique to LgtA and is not conserved in the Slt70 structure. This demonstrates that the bulgecin A is pliable, to fit different Lt active sites. The taurine part of the inhibitor is well supported in the active site of LtgA, with the sulphonated portion largely accessible by solvent.

### 2.3. Bulgecin A Inhibits LtgA, Slt70, and H. pylori Slt

**Figure 3:**
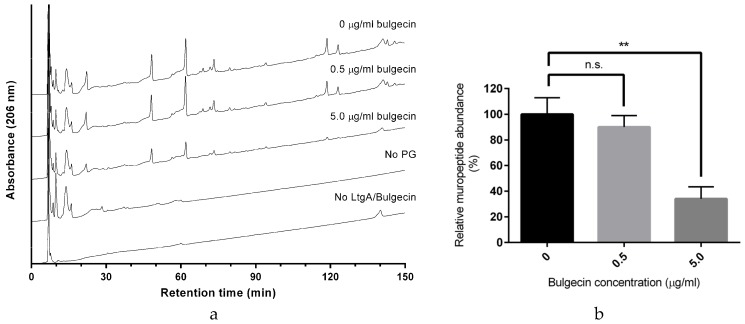
Inhibition of LtgA activity by Bulgecin A. (**a**) Reversed-phase (RP)-HPLC analysis of muropeptides released by LtgA digestion in the presence or absence of bulgecin A (0 μM, 9 μM, and 90 μM); (**b**) Proportion of muropeptides released in the presence of bulgecin A relative to that released in the uninhibited reaction. ** *p* < 0.01 (unpaired *t*-test).

**Figure 4:**
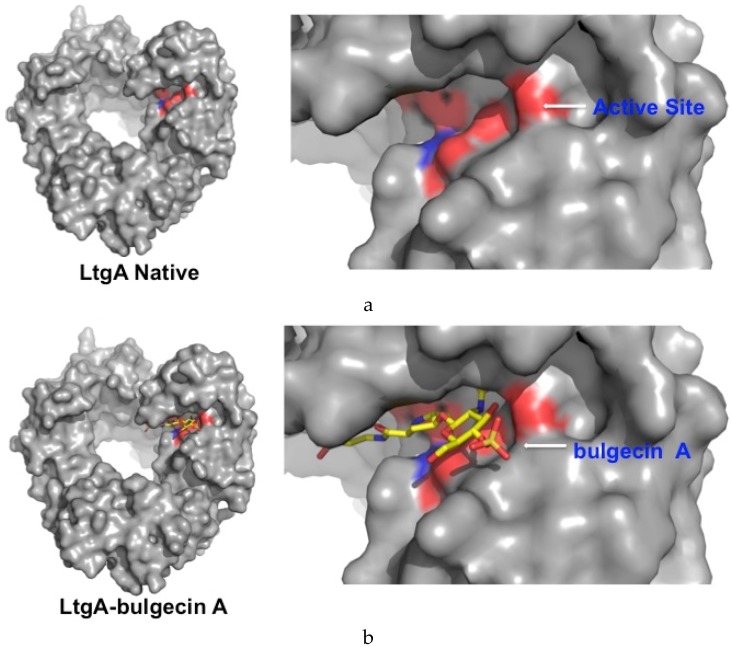
Surface model of LtgA native and LtgA in complex with bulgecin A. (**a**) Conserved residues that align the active site are highlighted in red; (**b**) Bulgecin A binds to the conserved active site groove.

Bulgecin A is an effective inhibitor against LtgA. The inhibitory potential of bulgecin A toward Lts was first observed with *E. coli* Slt70. The mode of inhibition by bulgecin A, as suggested by the Slt70-bulgecin A complex, is consistent with competitive inhibition [35]. It is bound in the substrate-binding site of LtgA and would interfere with binding of the PG glycan strand (Figure 3). The inhibitory potential of bulgecin A toward LtgA was relative to the inhibitor concentration; an increase in the concentration of the bulgecin A results in a comparable decrease in the enzymatic activity of LtgA. Sixty-six percent (±9.4%) of the activity of LtgA was lost in the presence of 90 μM of bulgecin A (A >60-fold excess of bulgecin A relative to LtgA; Figure 3). This supports the inhibitory potential of bulgecin A toward LtgA in vitro. Previous studies showed that bulgecin A primarily targets Slt70, the soluble Lt of *E. coli* [33,35]. The conservation of the active site of Lts can be exploited for future drug development because bulgecin A is bound in a conserved active site groove that is aligned with conserved residues (Figure 4a,b and Figure S5).

### 2.4. The Effects of Bulgecin A on Viability of Neisseria sp.

β-lactams are the antibiotic of choice for the treatment of *N. meningitidis* and *N. gonorrhoeae*, known as the pathogenic *Neisseria*. However, strains of *N. meningitidis* and *N. gonorrhoeae* have shown a reduced susceptibility to β-lactams such as penicillin G and amoxicillin. Although the treatment with these two antibiotics can still be effective; the treatment doses have to be increased drastically because lower doses may lead to treatment failure [39]. The primary mechanism of reduced susceptibility to β-lactams involves polymorphism of *penA*, the gene that encodes PBP2 in *Neisseria* sp. In the past, bulgecin A has been shown to improve the efficacy of β-lactams in *H. pylori* and *E. coli* [33,34]. We extended these observations to pathogenic *Neisseria* sp. and showed that a combination treatment of bulgecin A with β-lactams can improve efficacy of β-lactams against pathogenic *Neisseria* sp. with reduced susceptibility to penicillin, i.e., Pen^I^. We performed a basic E-test in the presence or absence of bulgecin A, with three β-lactam antibiotics (penicillin G, amoxicillin, cefotaxime) and used chloramphenicol, an antibiotic that targets protein synthesis, as a control. We prepared agar plates with four different concentrations of bulgecin A (0, 19, 38, or 75 µg/mL; Table 2). Bulgecin A restored the efficacy of penicillin G and amoxicillin of *N. meningitidis* and *N. gonorrhoeae*, and lowered the minimal inhibitory concentrations (MIC) of cefotaxime against *N. meningitidis* but had little impact on the MIC of chloramphenicol (Table 2). In addition, bulgecin A in combination with β-lactams was ineffective against β-lactamase-producing resistant strain 24753 TEM-1, which strongly supports a bactericidal synergism between β-lactams and bulgecin A that may target the Slt-PBP protein complex simultaneously. It is well established that Lts and PBPs form protein complexes [18,25,28,40,41,42]. Most notable are the interaction between Slt70 and PBPs 1b, 1c, 2, and 3 [43]. Targeting the Slt and PBPs for future antibiotic development could potentially define a new generation of antibiotic with dual targets that are synergistic.

## 3. Methods and Materials

### 3.1. Protein Expression and Purification

The recombinant LtgA protein construct was created using the primers listed below and standard molecular biology techniques. The constructs used in this study were Gluthatione S-Transferase (GST)-fusions from pGEX-4T1 (GE Lifesciences). The native LtgA protein was expressed in BL21 (DE3) Gold competent cells (Novagen) and grown in Luria Broth. Recombinant LtgA protein was induced with 0.6 mM isopropyl β-d-1-thiogalactopyranoside (IPTG) at an OD_600_ of 0.7–0.8 and harvested after 4 h of induction at 18 °C. After glutathione affinity chromatography and thrombin cleavage, proteins were purified to homogeneity by size exclusion chromatography (Superdex-200, GE Lifesciences) in 50 mM Hepes pH 7.4, 150 mM NaCl, and 1 mM β-mercaptoethanol (BME). After gel filtration, proteins were immediately used for crystallization.

### 3.2. Primer List

*ltgA* Forwardcgggatccacactgccagccggcaagaccccggc*ltgA* Reversecgaattctcagcgtgcaggaacaatgcccatacgc

### 3.3. Analysis of Recombinant LtgA and LtgA-GST Activity by Reversed-Phase HPLC

LtgA activity was assessed using purified PG from *N. meningitidis* strain 27256 as a substrate. PG was purified as described in [44]. The PG (100 µg) was incubated in the presence of 0 µM, 9 µM, or 90 µM of bulgecin A, in 12.5 mM sodium phosphate buffer pH 5.6. The reaction mix was preincubated at 37 °C for 5 min, then the reaction was initiated by the addition of 1.4 μM LtgA. Control reactions lacking PG or enzyme/inhibitor were also included. Total reaction volume was 200 µL. Reactions were performed in triplicate. Following overnight incubation at 37 °C, the reaction was stopped by incubating the samples in a boiling water bath for 3 min. Following centrifugation at 16,000 *g* for 10 min, the supernatant containing the soluble 1,6-anhydro-muropeptides was collected and analyzed by reversed-phase HPLC using a Shimadzu LC-20 system with a Hypersil GOLD aQ column (5 μm particle size, 250 × 4.6 mm, flow rate 0.5 mL/mL at 52 °C; Thermo Fisher Scientific (Waltham, MA, USA). The mobile phase gradient was 50 mM sodium phosphate pH 4.3 to 75 mM sodium phosphate pH 4.9 with 15% Methanol over 135 min. Muropeptide abundance was calculated as the sum of all peak areas above a baseline threshold value (20,000 units), using Shimadzu LCsolution software. We excluded peaks that appeared in the negative control reactions lacking PG, enzyme, or inhibitor, since these are not attributed to muropeptides.

### 3.4. The Effects of Bulgecin A on Viability of Neisseria Species

Etest^®^ (Biomérieux, Marcy l’Etoile, France) were carried out in duplicate by plating a lawn of the relevant *Neisseria* species onto Giolitti-Cantoni Broth (GCB) agar with Kellogg supplements plus [45] 0, 19, 38, or 75 µg/mL bulgecin A. Etest^®^ analyses were performed for three β-lactams: penicillin G, amoxicillin, and cefotaxim. Chloramphenicol was used as a non- β-lactam control. Three strains were used in this study, (1) *N. gonorrhoeae* Strain 24753 TEM-1; (2) *N. gonorrhoeae* Strain 24970 Pen^I^; (3) *N. meningitidis* Strain 28671 Pen^I^.

### 3.5. X-ray Crystallography

Crystallization screening was carried out by the sitting drop vapor diffusion method with a Mosquito^®^ (TTP Labtech) automated crystallization system. All crystals were grown at 18 °C using the hanging-drop vapor diffusion method. Crystals of LtgA were grown at 18 °C and appeared within 2–3 days. The concentration of LtgA was between 20–25 mg/mL. LtgA was crystallized in 1:1 (v/v) ratio against a well solution of 1.5–2.0 M ammonium sulfate, 0.1 4-Morpholineethanesulfonic acid buffer (MES) buffer pH 6.5 or 1.5–2.0 M ammonium sulfate, 0.1 M 2% (v/v) polyethylene glycol (PEG) 400 0.1 M Hepes pH 7.5. Crystals were rectangular in shape and grew to about 200–300 microns in length. To generate LtgA-bulgecin A complex, the native crystals of LtgA were soaked with a 10-fold molar excess of bulgecin A for 3 h and flash-cooled. Crystals were cryoprotected in a mixture of 50% Paratone and 50% Paraffin oils and flash-cooled in liquid nitrogen.

Phasing by LtgA-Bulgecin A complex was accomplished by molecular replacement using LtgA Native (4YIM) Phenix [46]. Building was performed using Coot [47], and restrained refinement was carried out using a combination of Phenix and a ccp4 software suite [46,48]. Molprobity was used during building and refinement for iterative structure improvements [49]. All structural figures were generated with Pymol (the PyMOL Molecular Graphics System, Version 1.5, Schrödinger, LLC, Cambridge, UK). The crystallographic parameters, data statistics, and refinement statistics are shown in Table 1. Coordinates and structure factors of LgtA-bulgecin A and LgtA mutant have been deposited in the Protein Data Bank (PDB) with the accession codes 5MPQ.

### 3.6. Docking Studies

The LtgA-chitopentaose complex was created using the MltE-chitopentaose (Protein Data Bank (PDB) ID: 4HJY) as a scaffold. Alignment of LtgA to MltE chitopentaose was done in Coot [47]. The full LtgA-chitopentaose complex was refined against the structure factors of MltE-chitopentaose complex using the ccp4 software, refmac [47]. The complex was examined and further positioned in Coot to facilitate chemical and geometric accuracy.

### 3.7. Sequence Alignments

The c-domain of LtgA and *E. coli* Slt70 were aligned with DNAstar Lasergene software (DNAstar, Madison, WI, USA) and its MegaAlign module using Clustal W.

## 4. Conclusions

Since antimicrobial resistance is a serious global threat, it is time to pursue different therapies to eradicate the most dangerous pathogens. The over-dependence on β-lactams is a key contributor to the rise of drug-resistant strains. LtgA is conserved across all species of *Neisseria*. *N. gonorrhoeae* LtgA shares a 98 percent similarity to *N. meningitidis* LtgA. There are successful vaccines that target *N. meningitidis*, however, as there are no available vaccines to fight *N. gonorrhoeae* infection, treatment has relied solely on antibiotic therapies. There is an increasing emergence of drug resistance in *N. gonorrhoeae* that underlines the need for new therapeutic molecules. Indeed, *N. gonorrhoeae* with resistance to all antibiotics used currently for treatment (super bug) are anticipated [44]. Our study demonstrates that *N. meningitides* susceptibility to β-lactams is restored in the presence of bulgecin A. The clinical breakpoint for penicillin G recommended by the European monitoring group (EMGM) on *N. meningitidis* is below 0.125 μg/mL for susceptibility, and greater than 1 μg/mL for resistance [39]. Already, the lowest concentration of bulgecin A (19 μg/mL) restored the susceptibility of *N. meningitidis* strain 28,671 Pen^I^ to below the susceptibility break-point (0.047 μg/mL). So far, β-lactams show improved efficacy in the presence of bulgecin A against *H. pylori*, *E. coli*, *P. aeruginosa*, *A. baumannii,* and now *N. meningitidis* and *N. gonorrhoeae.* The high-resolution structure of LtgA in complex with bulgecin A can be used to rationally design inhibitory molecules that target Lts in general. We propose the use of bulgecin A in combination with β-lactams as a new therapeutic strategy to fight antimicrobial resistance.

## Figures and Tables

**Table 1:** Data-collection and refinement statistics.

Data Collection	LtgA-Bulgecin A
Ligand Added	Bulgecin A
Data collection	
Wavelength (Å)	0.9795
Resolution range (Å)	45.84–1.78 (1.567–1.513)
Space group	*P2_1_2_1_2_1_*
Unit-cell parameters	
*a* (Å)	65.83
*b* (Å)	71.56
*c* (Å)	121.12
σ (°), β (°), γ (°)	90
Total reflections	108,346
Unique reflections	54,379
Multiplicity	2.0 (2.0)
Completeness (%)	98.00 (84.00)
Mean *I*/σ(*I*)	4.46 (0.67)
Wilson *B* factor (Å^2^)	21.65
*R*_merge_^†^	0.1064 (1.13)
Refinement	
*R*_factor_^‡^	0.2131 (0.3912)
*R*_free_ *	0.2570 (0.4084)
No. of atoms	4920
No. of waters	417
No. of protein residues	577
R.m.s.d., bonds (Å)	0.007
R.m.s.d., angles (°)	0.91
Ramachandran favored (%)	99
Ramachandran outliers (%)	1.0
*B* factors (Å^2^)	
Average	27.91
Macromolecules	27.43
Ligand	35.00
Solvent	33.54
All-atom clash score	2.09

Values in parentheses are for the outer shell. ^†^ *R*_merge_ = Σ_hkl_Σ_i_|*I*_i_(*hkl*) − 〈*I*_(*hkl*)_〉|/Σ_hkl_Σ_I_ *I*_(*hkl*)_; ^‡^ *R*_factor_ = Σ_hkl_ ||*F*_obs_ − *F*_calc_||/Σ_hkl_ |*F*_obs_|; * R_free_ was computed identically except that all reflections belonged to a test set consisting of a 10% random selection of the data.

**Table 2:** The effect of β-lactams combined with bulgecin A on *Neisseria* species with reduced susceptibitility to β-lactams. Minimal inhibitory concentrations (MICs) (µg/mL) of different antibiotics are shown, in the presence of 0–75 µg/mL bulgecin A.

***N. gonorrhoeae* Strain 24753 TEM-1**				
**Antibiotics**	**Bulgecin A (µg/mL)**			
	**0**	**19**	**38**	**75**
Penicillin G	4	4	4	3
Amoxicillin	16	12	16	12
Cefotaxim	0.003	0.002	0.002	0.002
Chloramphenicol	0.75	0.5	0.5	0.5
***N. gonorrhoeae* Strain 24970 Pen^I^**				
**Antibiotics**	**Bulgecin A (µg/mL)**			
	**0**	**19**	**38**	**75**
Penicillin G	0.5	0.094	0.094	0.047
Amoxicillin	0.75	0.38	0.250	0.19
Cefotaxim	0.125	0.047	0.047	0.047
Chloramphenicol	1.5	1.5	1	1
***N. meningitidis* Strain 28671 Pen^I^**				
**Antibiotics**	**Bulgecin A (µg/mL)**			
	**0**	**19**	**38**	**75**
Penicillin G	0.25	0.095	0.064	0.047
Amoxicillin	0.75	0.40	0.40	0.25
Cefotaxim	0.125	0.064	0.064	0.064
Chloramphenicol	0.5	0.5	0.4	0.5

## Supplementary Materials

The following are available online at http://www.mdpi.com/2079-6382/6/1/8/s1, Figure S1: (**a**) Ribbon model of LtgA highlighting the conserved U (green), L (orange), and C (grey) domains. The catalytic residue of LtgA E481 is shown in black; (**b**) Secondary structure of Slt70. The domain organization of LtgA represented by cylinders is close to Slt70, the soluble lytic transglycosylase from *E. coli*, Figure S2: The final F_o_-F_c_ electron density map of bulgecin A contoured at 3 sigma. The final 2F_o_-F_c_ map of LtgA contoured at 2 sigma, Figure S3: The catalytic domain of lytic transglycosylases is highly conserved, Figure S4: Bulgecin A occupies the active site of LtgA, Figure S5: Sequence alignment of the C-domain of LtgA and Slt70.
