# Supplementary material for: Bulgecin A: The Key to a Broad-Spectrum Inhibitor That Targets Lytic Transglycosylases
Source: Antibiotics (Basel). 2017 Feb 22;6(1):8. doi: 10.3390/antibiotics6010008 (PMC5372988; doi:10.3390/antibiotics6010008)
Supplement: Supplementary file 1 [file antibiotics-06-00008-s001.pdf]

Supplementary Materials: Bulgecin A: The Key to a Broad-Spectrum Inhibitor That Targets Lytic Transglycosylases

Allison H. Williams, Richard Wheeler, Constance Thiriau, Ahmed Haouz, Muhamed-Kheir Taha and Ivo Gomperts Boneca

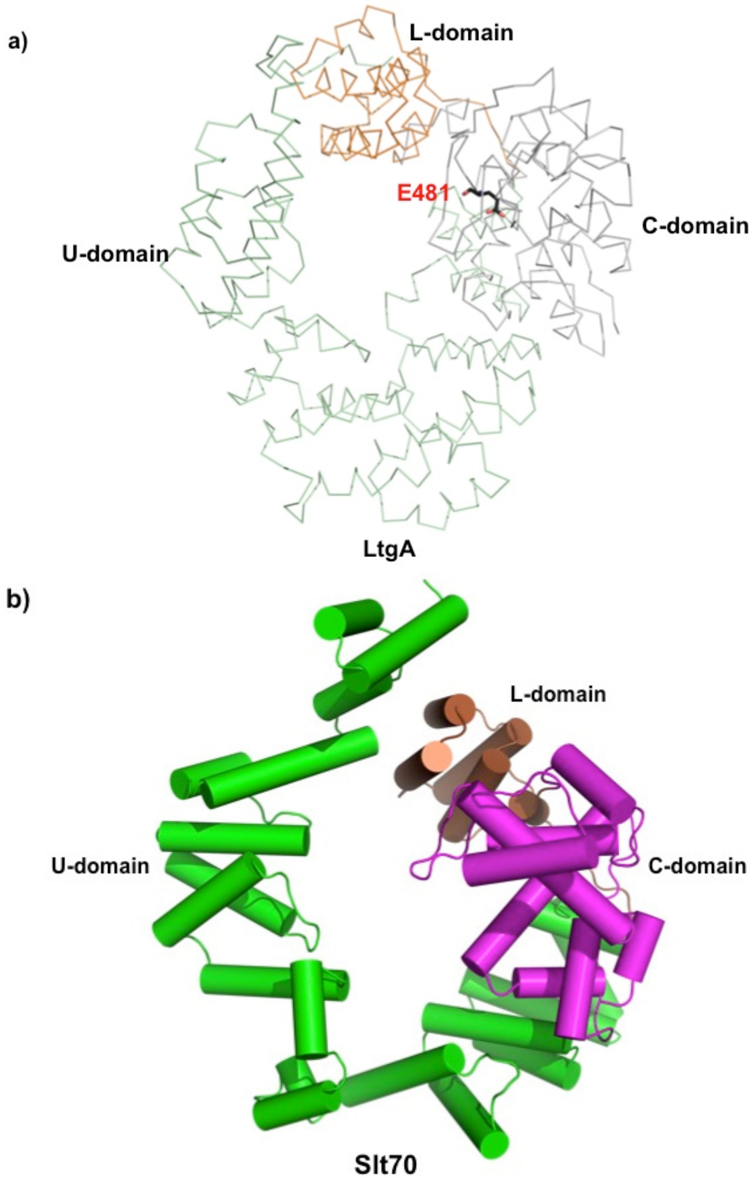

Figure S1. (**a**) Ribbon model of LtgA highlighting the conserved U (green), L (orange) and C (grey) domains. The catalytic residue of LtgA E481 is shown in black; (**b**) Secondary structure of Slt70. The domain organization of LtgA represented by cylinders is close to Slt70, the soluble lytic transglycosylase from *E. coli*.

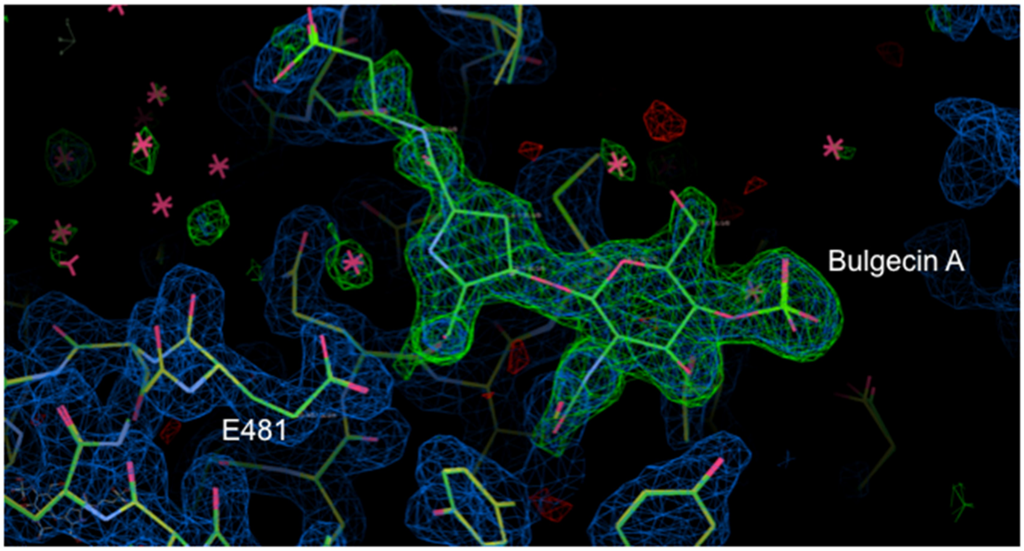

Figure S2. The final F_o-F_c electron density map of bulgecin A contoured at 3 sigma. The final $2F_o$-F_c map of LtgA contoured at 2 sigma.

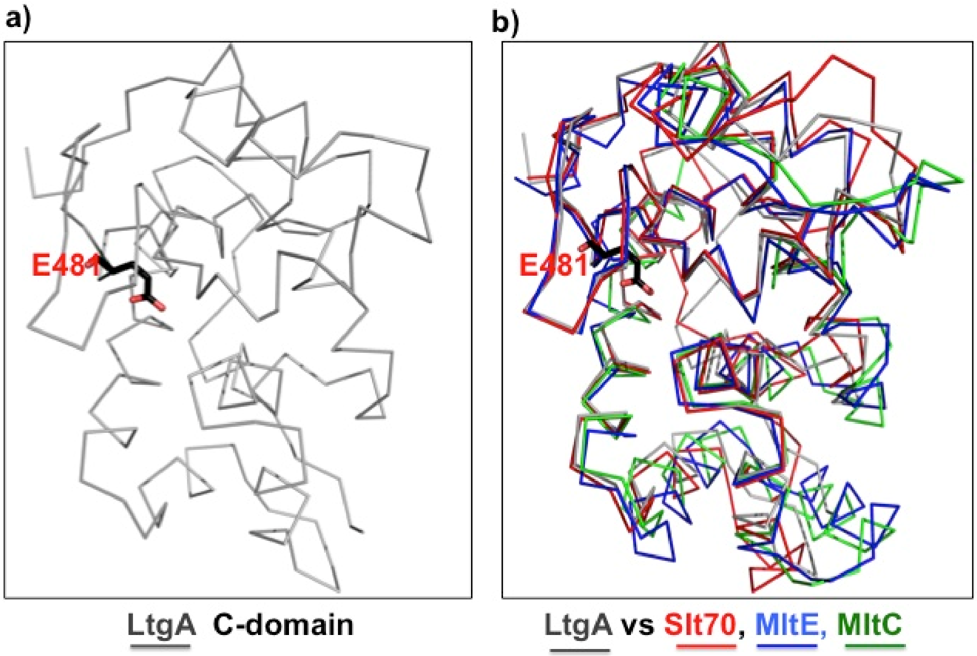

Figure S3. The catalytic domain of lytic transglycosylases is highly conserved. (**a**) Ribbon model of the catalytic domain of LtgA; (**b**) The catalytic domain of LtgA is aligned to Slt70, MltE, and MltC; all lytic transglycosylases from *E. coli*.

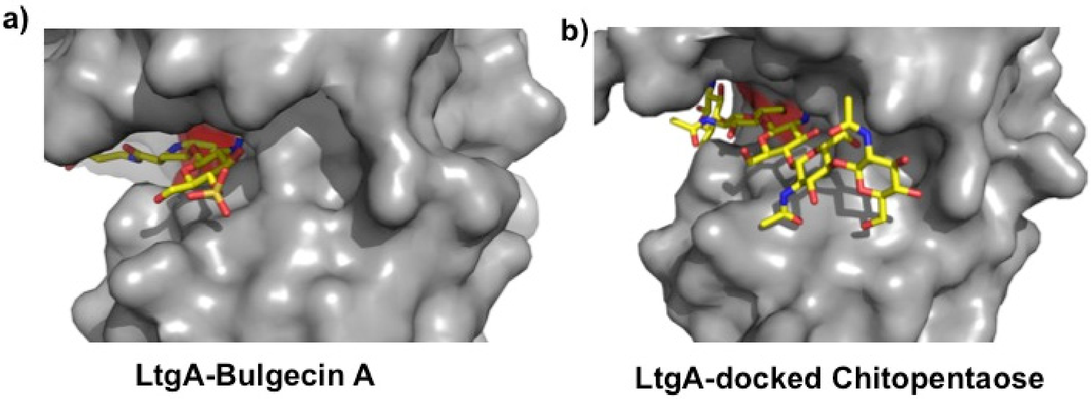

Figure S4. Bulgecin A occupies the active site of LtgA. (**a**) Surface model of the interaction between LtgA and bulgecin A. The model highlights the active site groove with bound bulgecin A. The catalytic residues are colored in red; (**b**) Surface model of docked chitopentaose in the active site groove of LtgA. Chitopentaose mimics the PG glycan strand.

```
Slt70 ------AFNNQWWDLSVQATIAGKLWDHLEERFPLAYNDLFKRYTSGKEIPQSYAMAIAR 476
LtgA  LTAAQTAFDHGFYDMAVNSAERTDRKLNYTLRYISPFKDTVIRHAQNVNVDPAWVYGLIR 479
            **:. ::*::*:::   .   .   *:   ::* . *::.  ::  ::. .: *

       ↓
Slt70 Q[illegible]SAWNPKVKSPVGASGLMQIMPGTATHTVKMFSIPGYSSPGQLLDPETNINIGTSYLQY 536
LtgA  QESRFVMGAQSRVGAQGLMQVMPATAREIAGKIGM----DAAQLYTADGNIRMGTWYMAD 535
      *** :   .:* ***.****:**.** . .  :.:    . .**   : **.:** *:

Slt70 VYQQFGNNRIFSSAAYNAGPGRVRTWLGNSAGRIDAVAFVESIPFSETRGYVKNVLAYDA 596
LtgA  TKRRLQNNEVLATAGYNAGPGRARRWQADT--PLEGAVYAETIPFSETRDYVKKVMANAA 593
      . ::: **.::::*.*******.* * .::   ::...:.*:******* ***:*:*  *

Slt70 YYRYFMGDKPTLMSATEWGRRY-- 618
LtgA  YYASLFGAPHIPLKQ-RMGIVPAR 616
      **  ::*     :.  . *
```

Figure S5. Sequence alignment of the C-domain of LtgA and Slt70. Residues predicted to be involved in glycan chain binding and/or catalysis are highlighted in red and those that are involved in PG stem peptide interactions are highlighted in blue.

© 2017 by the authors. Licensee MDPI, Basel, Switzerland. This article is an open access article distributed under the terms and conditions of the Creative Commons Attribution (CC BY) license (http://creativecommons.org/licenses/by/4.0/).
